# What Are the Trends in Women's Representation Among Principal Investigators of US Clinical Trials in the Field of Genetics?

**DOI:** 10.7759/cureus.75098

**Published:** 2024-12-04

**Authors:** Parmida Karimi, Asal Abolghasemi Fard, Aran Parnia, Radin Kamvar, Seyedehhasti Zojaji, Narges Behroozi, Amir Karimi

**Affiliations:** 1 Department of Dentistry, Herman Ostrow School of Dentistry, Los Angeles, USA; 2 Department of Cellular and Molecular Biology, Faculty of Modern Science and Technologies, Tehran Medical Sciences, Islamic Azad University, Tehran, IRN; 3 Department of Dentistry, University of California Los Angeles, Los Angeles, USA; 4 School of Medicine, University of Szeged, Szeged, HUN; 5 Department of Biomedical Sciences, Toronto Metropolitan University, Toronto, CAN; 6 Department of Engineering, University of Zanjan, Zanjan, IRN; 7 Department of Orthopedic Surgery, Case Western Reserve University School of Medicine, Cleveland, USA

**Keywords:** clinical trial, gender equity, genetics, leadership, research personnel

## Abstract

Introduction: Despite progress in the representation of women in the medical profession, substantial gender disparities persist in leadership roles, particularly in clinical trials. Clinical trials are crucial to evidence-based medicine, offering visibility, career advancement, and future funding opportunities for principal investigators (PIs). However, women remain underrepresented in these roles, especially in genetics. This study aimed to evaluate (1) the proportion of women PIs in genetic clinical trials and its change over time, (2) trial characteristics (phase, funding source, intervention type) associated with women PIs, and (3) the geographic distribution of women-led trials in the United States.

Methods: We analyzed 4,112 genetic clinical trials conducted in the United States and registered on ClinicalTrials.gov between 2007 and 2024. Trials lacking defined phases or complete investigator information were excluded. Statistical analyses were conducted using Fisher's exact tests and univariate linear regression.

Results: Women comprised 1,552 out of 4,112 (37.7%) PIs, with representation fluctuating from seven out of 24 (29.2%) in 2008 to a peak of 17 out of 29 (58%) in 2010 before leveling off to 573 out of 1,712 (33%) in 2024. Women PIs conducted a higher number of behavioral (240 out of 1,552, 15.5%) and other interventions (218 out of 1,552, 14%) studies, compared to 196 out of 2,560 (7.7%) and 222 out of 2,560 (8.7%), respectively, for men. On the other hand, men PIs had a higher number of genetic (111 out of 2,560, 4.3%) and drug (1,654 out of 2,560, 64.6%) trials, compared to 44 out of 1,552 (7.7%) and 841 out of 1,552 (54.2%), respectively, for women. Women received more federal funding (24 out of 1,552 (1.5%) vs. 17 out of 2,560 (0.6%)), while men dominated industry funding (695 out of 2,560 (27.1%) vs. 386 out of 1,552 (24.9%)).

Conclusion: Women remain underrepresented as PIs in genetic clinical trials, with no sustained growth over time. Mentorship programs, equitable funding policies, and increased visibility of women researchers are essential to address these disparities and foster equity in clinical trial leadership.

## Introduction

In recent decades, women have achieved substantial progress in entering the medical profession. Notably, in 2017, women medical school matriculants surpassed their men counterparts for the first time [1]. However, gender disparities persist in science and medicine. Numerous studies have identified a persistent achievement gap between men and women faculty in areas such as research productivity, career advancement, leadership roles, compensation, and scientific recognition [2-4]. Women researchers often offer distinct viewpoints, such as addressing health concerns specifically related to gender or prioritizing the inclusion of a diverse range of populations. This can result in outcomes that are more equitable and pertinent. The absence of their representation can lead to the continuation of biases and neglect of essential research areas, which ultimately restricts the significance and relevance of the results produced.

Clinical trials are integral to evidence-based medicine and critical for advancing new medical therapies and devices. Leading a clinical trial as a principal investigator (PI) provides physicians with enhanced visibility, national recognition, and opportunities for career progression, including promotions, tenure, and future funding [5,6]. Despite these benefits, women remain underrepresented as PIs in clinical trials. For example, a study of hip and knee arthroplasty clinical trials found that only two of 192 (1%) PIs were women from January 2015 to December 2021 [7]. Similarly, Nguyen et al. reported that from December 2014 to August 2019, just six of 266 (9.5%) cardiac surgery clinical trials were led by women [8].

Although these disparities are well documented in other specialties, the representation of women in genetic clinical trial leadership remains largely unexplored. Investigating the gender distribution of PIs in genetic clinical trials can provide valuable insights into the extent of this gender gap. This study aimed to address the following questions: (1) What proportion of genetic clinical trial PIs are women, and how has this changed over time? (2) Are certain trial characteristics (such as phase, status, funding source, or intervention) associated with women PIs? (3) What is the geographic distribution of genetic clinical trials led by women PIs within the United States?

## Materials and methods

Study design

We performed a thorough analysis of clinical trials using the publicly accessible ClinicalTrials.gov database, managed by the National Institutes of Health (NIH). This platform provides comprehensive data on registered trials, including information such as trial phase, sponsor, submission date, and location. In the United States, it is legally required to register all Phase II, III, and IV trials on ClinicalTrials.gov [8]. To focus on genetic-related clinical trials, we conducted a targeted search using specific Medical Subject Heading terms and keywords such as "genetics", "genetic testing", "gene therapy", "genomic sequencing", and similar terms. All trials found with these search terms were included in the screening process.

Data acquisition

On March 4, 2024, data from clinical trials were extracted in XML format from ClinicalTrials.gov and imported into Microsoft Excel (Microsoft Corporation, Redmond, Washington) for further processing. Our analysis focused on studies initiated in 2007 or later, aligning with the implementation of the US Food and Drug Administration Amendments Act. This legislation requires the registration of all Phase II, III, and IV trials conducted in the United States on ClinicalTrials.gov. Collected information included the trial phases (I, II, III, IV, or unspecified), sources of funding (industry, federal, NIH, or other), the year the trial started, types of interventions (behavioral, drug, device, procedure, etc.), the lead investigator's first name, and details of the trial's location (city, state, country). The US Census Bureau's definitions of Northeast, Midwest, South, and West were used for regional categorization.

PI gender identification

We compiled a list of investigators' first names using their full names and titles. Drawing from methods used in prior studies, we utilized Genderize.io (https://genderize.io), a well-known tool for determining gender from first names [9,10]. Genderize.io predicts gender based on a large dataset of names and their associated probabilities. Each first name was analyzed to provide a predicted gender with a probability value. In accordance with previous studies, names were categorized as man or woman, with a 60% threshold for gender assignment to ensure a balance between accuracy and inclusivity [7,9]. Mapping PI names to Genderize.io posed several challenges, particularly ambiguous or unisex names. Before entering names into Genderize.io, we manually extracted the first name of each PI from our XML file, which was a time-intensive process. The tool itself relies on probabilistic assignments based on name patterns, leading to inaccuracies for uncommon, culturally specific, or unisex names. Additionally, names with regional or linguistic variations often resulted in low confidence scores. Names with lower probabilities or inconclusive results were excluded.

Selection of clinical trials

**Figure 1:**
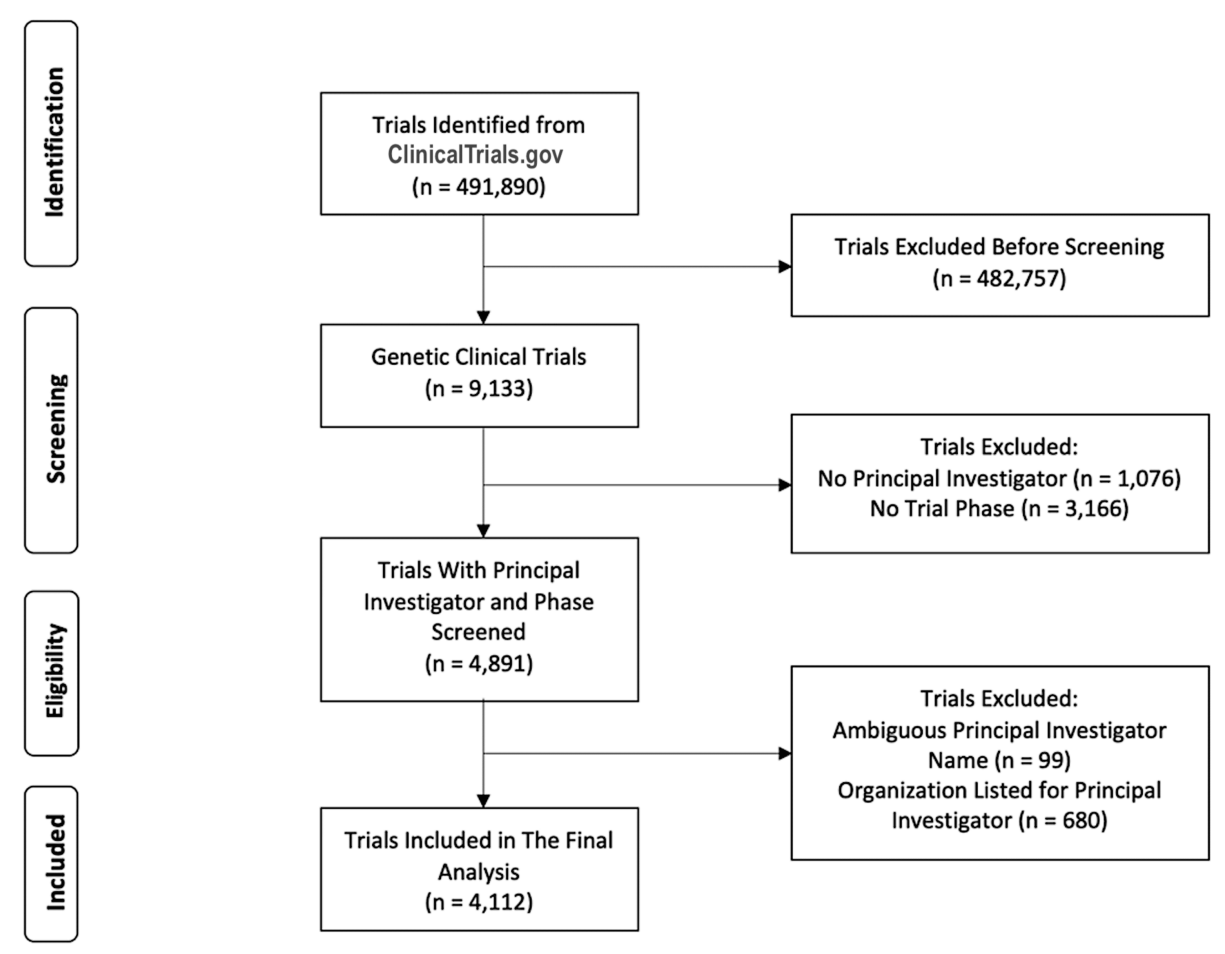
Diagram representing the screening process

Our study initially included 9,133 genetic trials conducted in the United States, drawn from the ClinicalTrials.gov database. To narrow our analysis, we selected only trials that had a PI listed and had a defined trial phase as per FDA standards (ranging from Phase I to Phase IV). Trials without a defined phase, those with organizations as PIs, or those with incomplete or unclear PI names were excluded as per previous studies [10,11]. We focused on trials with a defined phase to ensure consistency and facilitate meaningful comparisons. Phase designation reflects the structured progression of clinical trials, aligning with FDA standards. Including non-phase-based studies could introduce variability, making analysis less reliable, so narrowing the scope helped maintain clarity and comparability. After applying these criteria, 4,112 genetic trials were retained for the final analysis (Figure 1).

Outcomes

Our primary goal was to analyze the percentage of women PIs in genetic clinical trials and how this figure has changed over time. We compared the proportion of women PIs in genetic trials for each year from 2007 to 2024. To ensure accuracy, each trial was included only in the first year it was registered.

In addition, our secondary objectives were to examine the geographic distribution of woman PIs across the United States and investigate potential associations between trial characteristics and women PIs. Specifically, we looked at the proportion of women PIs across four key trial factors: trial phase, study status, funding source, and type of intervention.

Statistical methods

We presented the basic characteristics of the trials using frequencies and percentages for categorical variables such as geographic region, study phase, funding source, study status, and trial year. Fisher's exact test was used to assess the association between gender and categorical trial attributes, such as geographic region, study phase, funding source, and study status. This method was chosen because it provides accurate results for categorical data, even when some categories have low frequencies, as observed in certain subsets of our data. By applying this test, we ensured a robust analysis of gender differences across trial characteristics, maintaining the validity of our findings. Drawing from methodologies used in prior research, we employed univariate linear regression to analyze trends in the annual percentage of woman PIs [11]. We used heat maps with color gradients to visualize geographic variation. All statistical analyses were conducted using RStudio software version 2023.12.1 (Posit, Boston, Massachusetts), with statistical significance set at p < 0.05. Mapping was performed using QGIS 3.36.0.

## Results

The proportion of women PIs over time

**Figure 2:**
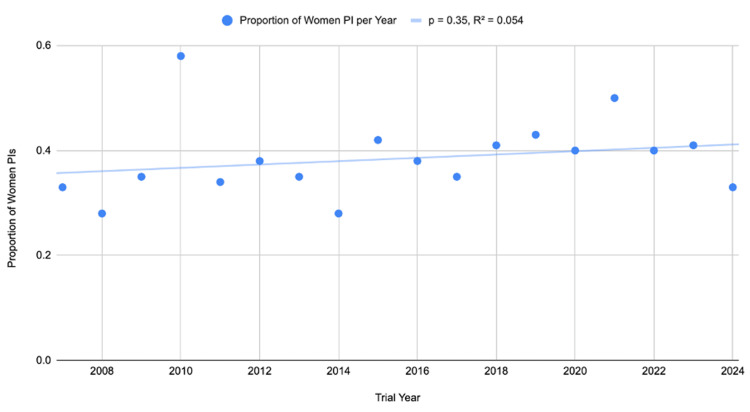
Women's representation as PIs of clinical trials over time PIs: principal investigators

The overall number of women serving as PIs was 1,552 out of 4,112 (37.7%) leadership roles held by women. This proportion fluctuated over the study period but showed no consistent upward or downward trend (p = 0.35, R^2^ = 0.054). The number of women leading clinical trials ranged from seven out of 24 (29.2%) in 2008 to a peak of 17 out of 29 (58%) in 2010 before leveling off to 573 out of 1,712 (33%) in 2024, as outlined in Table 1 and Figure 2.

**Table 1:** Distribution of women and men PIs over time PIs: principal investigators

Trial Year	Proportion of Women PIs, N (%)	Proportion of Men PIs, N (%)
2007	4 (40%)	6 (60%)
2008	7 (29.2%)	17 (17.8%)
2009	11 (35.5%)	20 (64.5%)
2010	17 (59.6%)	12 (41.4%)
2011	16 (35.8%)	30 (65.2%)
2012	19 (38%)	31 (62%)
2013	18 (35.3%)	33 (64.7%)
2014	20 (28.2%)	51 (71.8%)
2015	48 (42.1%)	66 (57.9%)
2016	47 (39.5%)	72 (61.5%)
2017	95 (36.6%)	172 (64.4%)
2018	126 (41.2%)	180 (58.8%)
2019	121 (43.2%)	159 (56.8%)
2020	106 (39.7%)	161 (60.3%)
2021	135 (50.4%)	133 (49.6%)
2022	105 (40.2%)	156 (59.8%)
2023	84 (41.6%)	118 (58.4%)
2024	573 (33.5%)	1139 (66.5%)

Women's representation and trial characteristics

The distribution of trial phases showed significant differences between men and women PIs (p < 0.0001). Trials with no applicable phase had the highest representation of women leaders, with 604 out of 1,552 (38.9%), compared to 666 out of 2,560 (26%) for men leaders. For trials with specified phases, Phase II had the highest representation of women leaders, with 393 out of 1,552 (25.3%), whereas Phase IV had the lowest, with 82 out of 1,552 (5.3%) of women PIs. Similarly, the highest percentage of men PIs was in Phase II, with 821 out of 2,560 (32.1%) (Table 2).

**Table 2:** Characteristics of clinical trials ^a^Other includes funding sources from individuals, universities, and organizations.

Characteristics	Men N (%) (n = 2560)	Women N (%) (n = 1552)	p-value
Status			<0.0001
Completed	1294 (50.5%)	768 (49.5%)	-
Active/Recruiting	709 (27.7%)	516 (33.2%)	-
Discontinued	439 (17.1%)	193 (12.4%)	-
Other	118 (4.6%)	75 (4.8%)	-
Study Phase			<0.0001
I	585 (22.9%)	314 (20.2.%)	-
II	821 (32.1%)	393 (25.3%)	-
III	322 (12.6%)	159 (10.2%)	-
IV	166 (6.5%)	82 (5.3%)	-
Not Applicable	666 (26%)	604 (38.9%)	-
Funding Source			0.003
Industry	695 (27.1%)	386 (24.9%)	-
Federal	17 (0.6%)	24 (1.5%)	-
NIH	178 (7%)	97 (6.3%)	-
Other	1670 (65.2%)	1045 (67.3%)	-
Intervention			<0.0001
Procedure	58 (2.3%)	25 (1.6%)	-
Device	148 (5.8%)	97 (6.3%)	-
Drug	1654 (64.6%)	841 (54.2%)	-
Behavior	196 (7.7%)	240 (15.5%)	-
Other^a^	222 (8.7%)	218 (14%)	-
Genetic	111 (4.3%)	44 (2.8%)	-
Biology	171 (6.7%)	87 (5.6%)	-
US Census Region			0.062
Midwest	360 (14.1%)	214 (13.8%)	-
Northeast	492 (19.2%)	318 (20.5%)	-
West	837 (32.7%)	492 (31.7%)	-
South	871 (34%)	528 (34%)	-

There was also a statistically significant difference in trial status between men and women PIs (p < 0.0001). A higher proportion of women PIs led active or recruiting trials, with 516 out of 1,552 (33.2%) compared to 709 out of 2,560 (27.7%) for men. Conversely, more men PIs led discontinued trials, with 439 out of 2,560 (17.1%) compared to 193 out of 1,552 (12.4%) for women PIs​ (Table 1).

There was a statistically significant difference in funding sources between men and women PIs (p = 0.003). Women PIs received a higher percentage of federal funding, with 24 out of 1,552 (1.5%), compared to 17 out of 2,560 (0.6%) for men PIs. Additionally, women PIs had a higher percentage of studies funded by other sources, with 1,045 out of 1,552 (67.3%), compared to 1,670 out of 2,560 (65.2%) for men PIs. In contrast, a slightly higher percentage of men PIs received industry funding at 695 out of 2,560 (27.1%), compared to 386 out of 1,552 (24.9%) for women PIs​ (Table 2).

There was a significant difference in the distribution of intervention types between men and women PIs (p < 0.0001). Women PIs led a larger proportion of studies involving behavioral interventions, with 240 out of 1,552 (15.5%), and other interventions with 218 out of 1,552 (14%), compared to their men counterparts, who had 196 out of 2,560 (7.7%) and 222 out of 2,560 (8.7%), respectively. On the other hand, men PIs had a higher proportion of studies involving genetic interventions, with 111 out of 2,560 (4.3%), and drug interventions, with 1654 out of 2,560 (64.6%), compared to 44 out of 1,552 (2.8%) and 841 out of 1,552 (54.2%) for woman PIs​ (Table 2).

Geographic distribution of women PIs

**Figure 3:**
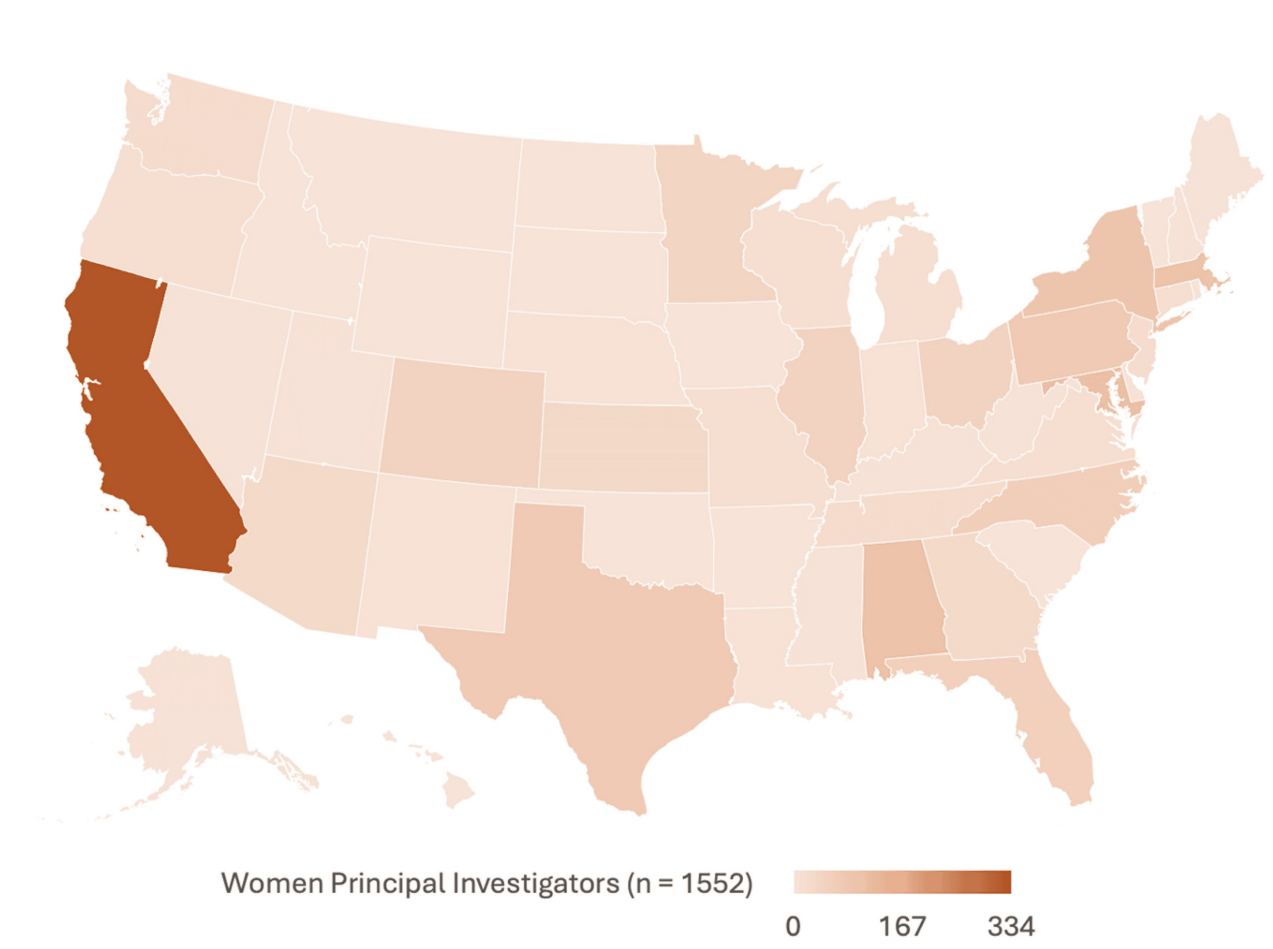
Geographical distribution of women PIs PIs: principal investigators

**Figure 4:**
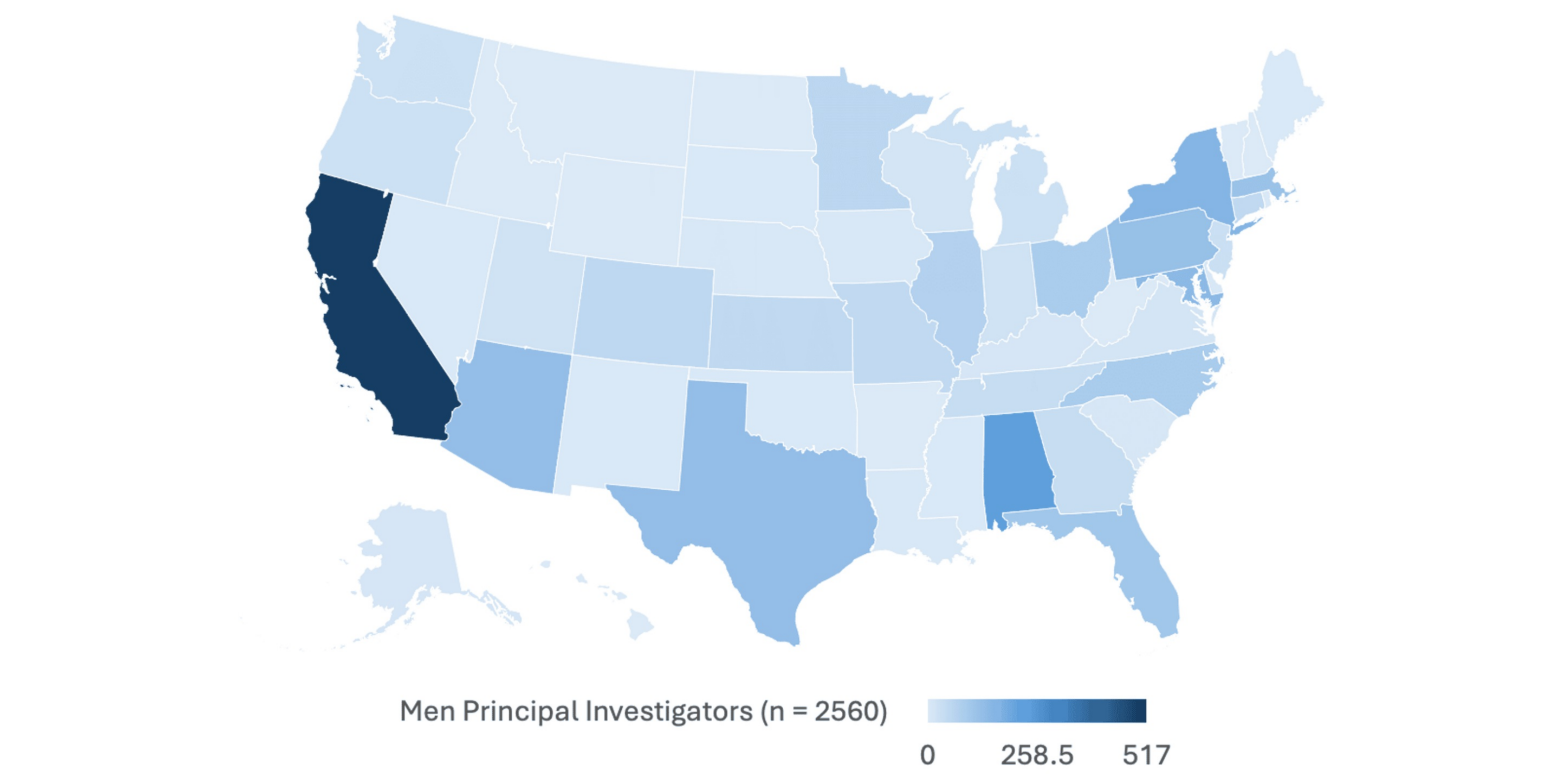
Geographical distribution of men PIs PIs: principal investigators

There was no significant difference in the geographic distribution of trials led by men and women PIs (p = 0.062). The proportion of women PIs was relatively similar across US Census regions to that of their male counterparts. The states with at least 10 trials that had the highest proportions of women PI were New Mexico (7 of 10, 70%), the District of Columbia (33 of 66, 66%), Virginia (15 of 30, 50%), and Nebraska (7 of 14, 50%). Conversely, the lowest proportions of woman PIs were found in Kentucky (2 of 16, 13.3%), Utah (4 of 26, 15.4%), and Arizona (30 of 160, 18.9%) (Table 3) (Figures 3, 4).

**Table 3:** Distribution of men and women PIs across US states PIs: principal investigators

States	Women PIs (n = 1552)	Men PIs (n = 2560)
Alabama	108	229
Alaska	4	11
Arizona	30	130
Arkansas	0	0
California	334	517
Colorado	49	53
Connecticut	17	49
Delaware	2	5
District of Columbia	33	33
Florida	56	107
Georgia	29	40
Hawaii	0	0
Idaho	1	0
Illinois	50	72
Indiana	9	24
Iowa	1	5
Kansas	32	50
Kentucky	2	13
Louisiana	5	6
Maine	3	3
Maryland	112	144
Massachusetts	105	118
Michigan	17	28
Minnesota	44	59
Mississippi	2	2
Missouri	17	48
Montana	1	1
Nebraska	7	7
Nevada	2	4
New Hampshire	3	1
New Jersey	21	31
New Mexico	7	3
New York	91	160
North Carolina	61	92
North Dakota	0	1
Ohio	48	88
Oklahoma	1	2
Oregon	15	26
Pennsylvania	77	126
Rhode Island	1	4
South Carolina	1	8
South Dakota	0	3
Tennessee	22	41
Texas	75	133
Utah	4	22
Vermont	0	0
Virginia	15	15
Washington	22	28
West Virginia	2	1
Wisconsin	14	17
Wyoming	0	0

## Discussion

Clinical trial leadership is a prestigious role for academic physicians, offering opportunities for increased visibility, career advancement, and access to future funding. It also allows physicians to make substantial contributions to the medical field and improve patient outcomes. The representation of women in leadership roles within genetic clinical trials has not been thoroughly studied or characterized. Our findings revealed that while the proportion of women leading clinical trials fluctuated over time, there was no consistent upward or downward trend. Representation ranged from a low of seven out of 24 (29.2%) in 2008 to a peak of 17 out of 29 (58%) in 2010 before leveling off to 573 out of 1,712 (33%) in 2024. Notably, women surpassed men as PIs during only two years of the study period, making up only 1,552 out of 4,112 (37.7%) of the entire PIs of clinical trials. Women PIs were more likely to lead studies focusing on behavioral and other interventions, whereas men PIs led a higher proportion of genetic and drug trials. Additionally, women PIs received more funding from other sources, while men PIs were more likely to secure industry funding. These findings underscore the need for continued efforts to address gender disparities in clinical trial leadership and highlight the importance of developing strategies to promote equity in this field. Achieving sustained progress will require targeted policies, mentorship opportunities, and collaboration to create an inclusive research environment that supports diverse leadership.

Since 2007, there have been more than two times as many clinical trials led by men as women in genetics. The proportion of women leading clinical trials showed fluctuations over time but remained stagnant between 2007 and 2024, with women comprising 33% of PIs in both years. This is not consistent with women's representation as PIs in other medical specialties [9,12,13]. For instance, Burkhart et al. reported that the proportion of women leading orthopedic clinical trials increased from 13% in 2007 to 22% in 2022 [9]. One possible explanation for this discrepancy is that genetics began with a greater baseline of female PIs in 2007, which lessened the perceived urgency of equity initiatives. This early lead may have resulted in fewer measures focused on improving representation, as opposed to other fields that benefitted from focused campaigning to address disparities.

Furthermore, genetics may have received less institutional push to enhance leadership diversity, resulting in stagnation over time. Even though there was a higher proportion of women PIs in genetic trials as compared to other medical specialties, the lack of growth is concerning. Certain specialties have demonstrated substantial growth in women's representation, with women comprising the majority of clinical trial PIs in some cases. For example, the proportion of women PIs in Phase III gynecologic oncology trials increased markedly from 10% in 2010 to 60% in 2020 [11]. This suggests there is still substantial room for improvement. Women exceeded men as PIs in genetic clinical trials only in 2008 and 2010, suggesting isolated advances rather than a sustained trend toward gender parity in this discipline. These brief periods of female predominance might be attributed to external influences such as specific regulations, financial campaigns, or cultural transformations that emphasized women's leadership in science during those years. However, their brief existence shows that such attempts were either not sustained or insufficient to overcome larger systemic obstacles. To increase the number of women PIs in genetics, institutions and professional organizations should implement mentorship programs focused on career development and provide networking opportunities tailored to their needs. Continuous evaluation of clinical trial leadership is crucial as the presence of women in academic leadership roles within genetics evolves. Understanding these trends can guide the development of effective strategies to reduce gender disparities and promote equity in the field.

Our analysis revealed that women were more likely to receive federal or other funding, whereas men were predominantly funded by industry sources. This aligns with previous studies indicating that men receive a disproportionately larger share of industry funding. For instance, a 2016 study found that women academic orthopedic surgeons received only 29% of industry payments, even after adjusting for factors such as faculty rank, H-index, and subspecialty selection [14]. These funding disparities are not isolated to orthopedics but are widespread across medical specialties for women surgeon-scientists [8,11,15]. A potential explanation for this inequity is that 75% of physicians with financial ties to biomedical companies are men [16]. Funding differences between male and female PIs may impact trial scope and results. Women PIs' dependence on federal and "other" funding sources indicates a preference for fundamental or exploratory research, which may have less immediate economic appeal but contributes to scientific progress. Men PIs, on the other hand, may receive more industry funding, allowing them to conduct larger, commercially motivated investigations. These disparities may influence the types of research conducted and how findings are translated into clinical practice, emphasizing the importance of fair financing to promote a balanced research environment.

Interestingly, our findings differ slightly from those of Burkhart et al., who reported no significant differences in the distribution of men and women across trial phases, statuses, or funding sources in orthopedics [9]. The distinctions from Burkhart et al. might stem from the distinctive characteristics of genetics research, encompassing a variety of interventions such as gene therapy and diagnostics, which draws a wider array of researchers and funding opportunities. Genetics trials may additionally provide greater chances for exploratory or federally supported studies, where there is a higher representation of women, in contrast to orthopedics, which is generally more influenced by industry and consistent in trial attributes. Furthermore, our results showed an increased number of female PIs in Phase II trials, which might be attributed to the collaborative character of these investigations, which involve input from interdisciplinary teams and are consistent with leadership styles that emphasize cooperation and communication. Furthermore, Phase II trials frequently obtain funding from smaller organizations or agencies that encourage diversity, possibly increasing prospects for women-led research. These trials are usually carried out in academic or research-focused institutions, where women are more likely to be represented than in industrial settings, and they benefit from mentorship and support networks. Furthermore, the combination of clinical practice and research in Phase II studies may appeal to women who want to combine patient care with medical advancements. Additionally, our analysis demonstrated that women PIs led a higher proportion of studies focused on behavioral and other interventions, while men PIs were more involved in drug-related trials. This observation is consistent with other studies showing that women are more likely to lead observational studies [9,17].

Understanding the distribution of PIs across various trial phases and intervention types is critical for informing research strategies, guiding funding allocation, and shaping policy decisions to address gender disparities in clinical trial leadership. The lack of women in higher-phase trials, such as Phase III and IV, may stem from obstacles related to resources and career growth. These trials demand significant funding and leadership experience, which tend to be more readily available to men due to systemic issues such as implicit bias in funding, limited mentorship, and fewer opportunities for leadership. Such challenges restrict women's capacity to oversee extensive, resource-heavy studies, thereby sustaining the gender disparity in advanced clinical research.

A diversified strategy is needed to address the differences in clinical trial PIs between men and women in the field of genetics. Mentorship programs should be established by institutions and professional associations to assist and guide women who wish to pursue careers as PIs in genetics. To ensure fair representation, department heads must aggressively advance women faculty members to prominent roles in leadership and research. Grant programs aimed at women researchers should be created and extensively publicized in order to further address these inequities. Making successful women academics more visible through prizes, press attention, and high-profile conference speaking engagements can motivate and inspire others to follow in their footsteps. Equal funding distribution should also be required by institutional and governmental policy reforms, guaranteeing that women researchers have the tools necessary to conduct and complete clinical studies. These could also be integrated with efforts to minimize implicit bias in hiring and funding choices, guaranteeing women equal chances. Ultimately, instituting transparency standards regarding gender representation in leadership roles could ensure accountability for institutions while offering metrics for assessing progress toward equity. When these strategies are used together, they can foster a more equitable and inclusive research setting.

Our analysis has several limitations that should be considered when interpreting the findings. First, our reliance on data from ClinicalTrials.gov, a global registry for clinical trials, introduces a potential bias, as trials not requiring FDA regulation are not mandated to register, potentially underrepresenting international studies. Second, the use of Genderize.io to determine the gender of PIs carries inherent risks of misclassification, particularly for names with ambiguous or culturally diverse origins. Although we excluded names with lower prediction probabilities to improve accuracy, this approach may have inadvertently removed some correctly classified individuals, potentially affecting the representativeness of our findings. Additionally, our analysis was limited to gender as the sole diversity variable, excluding other important dimensions of social identity such as race, ethnicity, and socio-economic background. Finally, we did not provide a detailed breakdown of subspecialties within genetic trials or account for other potential confounding variables, such as trial initiation year, that may influence gender representation. Future studies should address these limitations by incorporating broader datasets, expanding diversity metrics, and utilizing software that can more accurately determine the gender of a name or directly contacting PIs to determine accurate gender to capture the intersectionality of social identity factors and their impact on clinical trial leadership.

## Conclusions

Our findings highlight significant gender disparities in clinical trial leadership within genetics, with women underrepresented as PIs and showing limited progress over time. While women were more likely to receive federal or other funding, men predominantly secured industry funding, reflecting broader inequities seen across medical specialties. Despite some fields achieving notable growth in women's representation, the standstill in genetics underscores the need for targeted interventions. Addressing these disparities will require a comprehensive approach, including mentorship programs, equitable funding policies, increased visibility for women researchers, and active promotion of women into leadership roles. By fostering an inclusive research environment and addressing funding imbalances, we can work toward achieving greater equity in clinical trial leadership, ultimately enhancing the diversity and impact of genetic research.
